# Mucosal distribution of somatostatin-secreting gastric Delta cells in children with gastrointestinal reflux diseases

**DOI:** 10.3389/fped.2023.1275842

**Published:** 2023-10-20

**Authors:** Dong-Uk Kim, Jae Yoon Na, Seung Sam Paik, Seungyun Jee, Young Ho Lee, Yong Joo Kim

**Affiliations:** ^1^ Graduate School of Medical Science and Engineering, Korea Advanced Institute of Science and Technology, Seoul, Republic of Korea; ^2^ Department of Pediatrics, Hanyang University College of Medicine, Seoul, Republic of Korea; ^3^ Department of Pathology, Hanyang University College of Medicine, Seoul, Republic of Korea

**Keywords:** somatostatin-secreting cell, children, stomach, duodenogastric reflux, helicobacter pylori

## Abstract

**Introduction:**

Gastric delta cells (D-cells) secrete somatostatin, which is the primary paracrine suppressor of acid secretion. The number and distribution of D-cells were investigated in children exhibiting endoscopic findings of duodenogastric and gastroesophageal reflux. This study aimed to determine whether the number of D-cells in the gastric body differs from that in the gastric antrum in children using endoscopic findings.

**Methods:**

We retrospectively used immunohistochemical assessments to determine the number of D-cells in the gastric body and antrum in 102 children who presented with abdominal symptoms. The number and distribution of D-cells were investigated according to symptoms, endoscopic findings of gastroesophageal reflux and duodenogastric reflux, and *Helicobacter pylori* infection status.

**Results:**

The average age of the patients was 13.3 ± 3.3 years, and the male-to-female ratio was 1:1.68. The mean number of D-cells per high-power field in the antrum and body did not significantly differ by symptoms. However, these values were significantly lower in the gastric body than in the antrum for all symptoms (*p* < 0.05). Children with reflux had a higher mean number of D-cells (9.6 ± 8.8) in the gastric body than did those without reflux (4.3 ± 3.4) (*p *= 0.007). Furthermore, the number of D-cells in the gastric body was marginally significantly lower in *Helicobacter pylori*-positive children (4.9 ± 6.5) than in *Helicobacter pylori*-negative children (8.5 ± 8.2) (*p* = 0.053).

**Conclusion:**

The number of D-cells in the gastric body decreased in *Helicobacter pylori*-positive children but significantly increased in children with duodenogastric reflux. Therefore, somatostatin peptide secretion by D-cells may be a major pathophysiological pathway in gastrointestinal reflux disease.

## 1. Introduction

Chronic abdominal pain is a common symptom in children. The prevalence of organic causes of chronic abdominal pain is approximately 5% in the general population and 40% in pediatric gastroenterologists' investigation (1). Diagnostic methods for differential diagnosis have been developed and applied over the last three decades. Gastrointestinal (GI) endoscopic examinations and pathological analyses may be helpful in the differential diagnosis of abdominal pain. Recently, the most serious GI diseases have been effectively diagnosed and managed. However, GI motility disorders tend to persist for a long time and easily recur. Reflux esophagitis and chronic gastritis are most commonly found on upper GI (UGI) endoscopy. Duodenogastric reflux (DGR) gastritis, also known as bile reflux gastritis, is occasionally observed during UGI endoscopy. DGR enhances the cytotoxicity of bile acids—leading to cell membrane damage—and changes the composition of the microbiota (2). The pathological findings of postgastrectomy DGR gastritis include antral foveolar hyperplasia, lamina propria edema, the infiltration by a few inflammatory cells, and vascular congestion (3). The pathological findings in these reflux diseases in adults have been well explained; however, the features of primary and symptomatic DGR in children have not been well documented. A study showed that foveolar hyperplasia and mucosal vascular congestion were the histological findings of primary DGR in children (4). We sought to determine the possible physiological role of GI peptides in the development of reflux diseases.

The defensive factors in the UGI mucosa are the mucus layer, epithelium, and blood supply. Factors that induce foregut mucosal injury include infections, medications, allergies, food composition, and vascular disorders (5). In healthy individuals, there is a delicate equilibrium between these defense mechanisms and injuries. The abundance of injury-causing factors disrupts this balance, leading to mucosal injury. Helicobacter pylori (H. pylori) is also known to induce peptic ulcers. Replacement of normal gland cells by inflammatory cells results in a change in pH, depending on the colonization site in the stomach (6, 7). Stomach acid secretion is regulated by paracrine, hormonal, and vagal factors. Various endocrine cells, such as gastrin cells (G-cells), enterochromaffin-like (ECL) cells, and somatostatin cells (D-cells), also play roles in regulating stomach acid secretion. G-cells release gastrin in the antrum, which triggers ECL cells to release histamine and activates parietal cells to secrete hydrochloric acid (8). D-cells release somatostatin, the primary paracrine inhibitor that controls gastric acid secretion (9). Somatostatin delays gastric emptying and increases stomach volume by regulating gastric motility (10). Somatostatin also suppresses acid and pepsin secretion, inhibits gastrin release (11), and reduces lower esophageal sphincter (LES) pressure (12). Antral somatostatin affects G-cells; however, oxyntic somatostatin acts on both parietal and ECL cells. Consequently, somatostatin suppresses gastric acid secretion via diverse pathways and remains localized within the stomach (13, 14).

Thus, we aimed to examine the distribution and number of D-cells in the gastric body and antrum in children with chronic abdominal symptoms associated with reflux esophagitis, DGR gastritis, and *H. pylori*-induced gastritis using immunohistochemical pathologic studies.

## 2. Materials and methods

### 2.1. Study design

The number and distribution of somatostatin-secreting D-cells in the gastric antrum and body of the stomach were investigated according to patient symptoms, GI endoscopic findings of reflux esophagitis and DGR, and the presence of *H. pylori* infection. We retrospectively analyzed the data of children who underwent D-cell immunohistochemical examinations.

### 2.2. Patients

We performed a retrospective study of children who visited the Department of Pediatrics at Hanyang University Hospital between June 2016 and May 2022. An UGI endoscopy was performed for children who presented with GI symptoms (abdominal pain, substernal pain, vomiting, and diarrhea). Patients diagnosed with inflammatory bowel disease, GI bleeding, hepatobiliary disease, and pancreatic disease were excluded. Endoscopic findings of reflux esophagitis were documented according to the LA classification of gastroesophageal reflux disease (GER) (15). The bile reflux findings included duodenogastric-esophageal reflux and DGR. The number of somatostatin-secreting D-cells in the gastric body and antrum of 102 children was investigated using immunofluorescence. The study protocol was approved by our Institutional Review Board (2022-06-005-003).

### 2.3. Immunohistochemical staining for D-cell detection

An endoscopic biopsy of the stomach was performed, and a primary rabbit polyclonal antibody against somatostatin (1:200, ab183855; Abcam, Cambridge, UK) was used to detect D-cells in gastric biopsy tissues. The tissue samples were prepared in 4-μm-thick sections on coated glass slides. Immunostaining was performed using a Bond-Max automated immunohistochemical staining machine (Leica Biosystems, Nussloch, Germany), according to the manufacturer's protocol.

**Figure 1:**
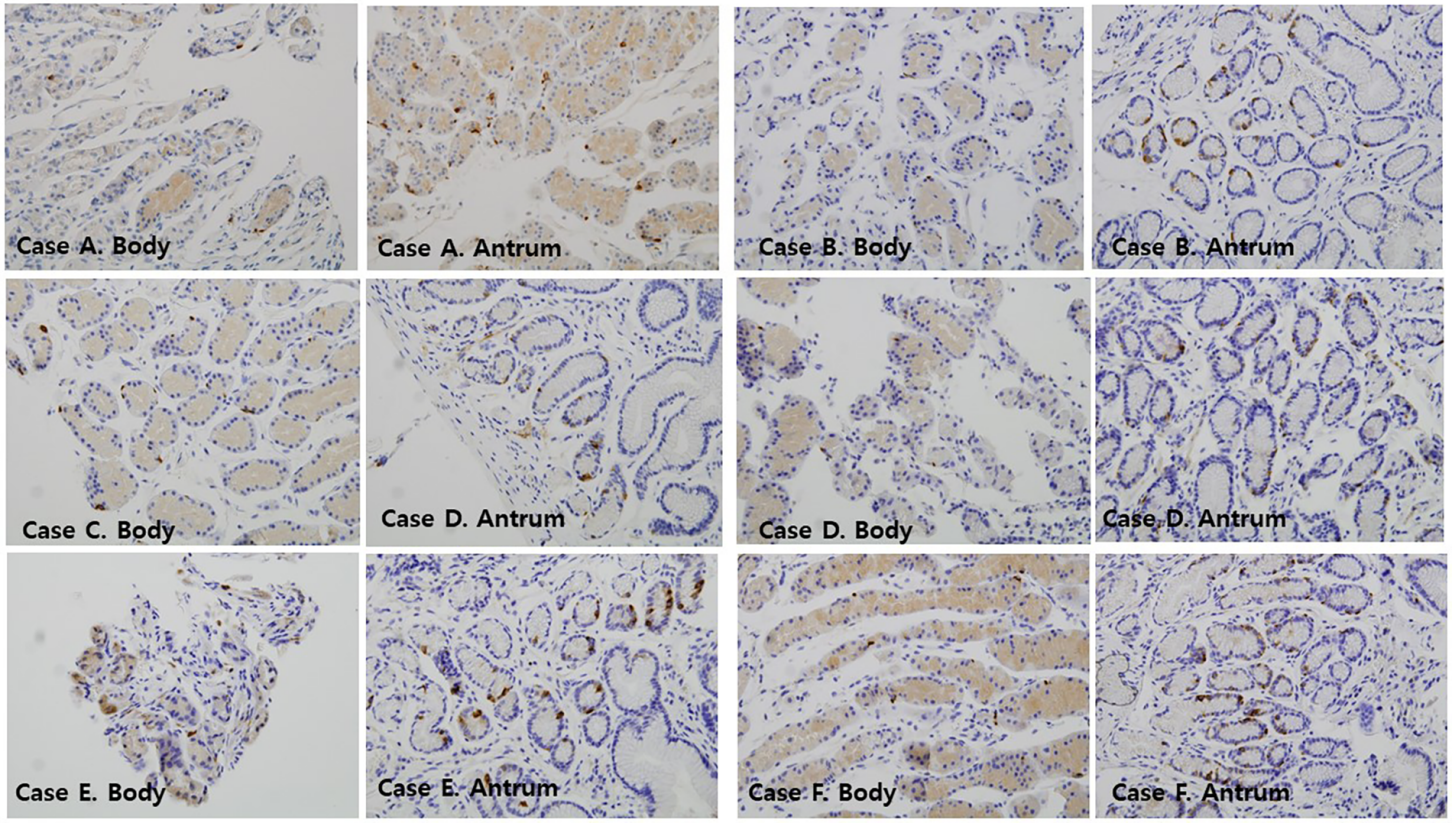
Immunofluorescence findings of somatostatin-immunoreactive D-cells in the mucosa of the gastric antrum and gastric body of the patients ( × 400). Case A: a 12-year-old female with GER; Case B: a 10-year-old female with DGER; Case C: a 14-year-old female with GER; Case D: a 16-year-old female with DGER; Case E: an 11-year-old female with no mucosal lesions; Case F: a 12-year-old female with no mucosal lesions. DGER, duodenogastric-esophageal reflux; GER, gastroesophageal reflux.

Two independent pathologists (SP and SJ) who were blinded to patients' clinical outcomes counted the somatostatin-positive D-cells using high-power microscopy (× 400 magnification). Representative photomicrographs showing positive somatostatin-secreting D-cells in the body and antrum of the stomach according to gastrofibroscopic findings are shown in Figure 1.

### 2.4. Statistical methods

Statistical analyses were performed using IBM SPSS Statistics (version 27.0) (IBM Corp., Armonk, NY, USA). Statistical significance was set at *p* < 0.05. The Mann–Whitney *U* and Kruskal–Wallis tests were used to identify differences between two and more than two groups, respectively. If the result of a test was significant, a *post-hoc* Tukey test was performed. All results are presented as means ± standard deviations (SD).

## 3. Results

### 3.1. Baseline characteristics of the patients

In total, 102 children were enrolled in this study (Table 1). The average age was 13.3 years, with a SD of 3.3 years. The male to female ratio was 1:1.68. The most common symptom was abdominal pain, occurring in 86.3% of children. Other symptoms included nausea and/or vomiting (31.4%), diarrhea (16.7%), and substernal pain (12.7%). Based on the endoscopic findings, 75.5%, 13.7%, 17.6%, and 12.7% of the children were diagnosed with gastritis, duodenitis, gastric ulcers, and duodenal ulcers, respectively. Reflux disease, GER, and bile reflux were found in 73.5%, 34.3%, and 39.2% of the patients, respectively. Ten percent of the patients had an active *H. pylori* infection. The number of D-cells per high-power field (HPF) in the gastric antrum (15.7 ± 12.1) was almost double that in the gastric body (8.2 ± 8.1).

**Table 1:** Baseline characteristics of patients.

Clinical characteristics	Participants (*n* = 102)
Age (year)	13.3 ± 3.3
Sex (male: female)	38:64 (1:1.68)
Clinical symptoms	
Substernal pain	13 (12.7%)
Abdominal pain	88 (86.3%)
Nausea and vomiting	32 (31.4%)
Diarrhea	17 (16.7%)
No symptom	4 (3.9%)
Endoscopic findings	
Gastritis	77 (75.5%)
Duodenitis	14 (13.7%)
Gastric ulcer	18 (17.6%)
Duodenal ulcer	13 (12.7%)
Reflux	75 (73.5%)
Reflux esophagitis	35 (34.3%)
Bile reflux (DGER and/or DGR)	40 (39.2%)
*Helicobacter pylori* infection	10 (10.0%)
Number of D-cells/HPF	
Antrum	15.7 ± 12.1
Body	8.2 ± 8.1

Values are presented as median (interquartile range) or mean ± SD. DGER, duodenogastric-esophageal reflux; DGR, duodenogastric reflux.

### 3.2. Number of D-cells per HPF in relation to patient symptoms

**Figure 2:**
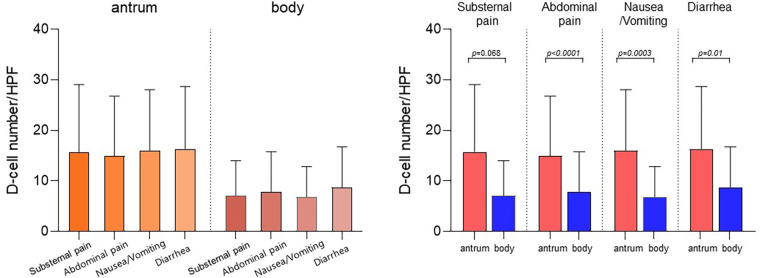
Number of D-cells in the antrum and body according to the symptoms of the patients. Children with substernal pain had 15.8 ± 12.8 D-cells per HPF in the gastric antrum and 7.1 ± 6.7 D-cells in the gastric body. In those with abdominal pain, the number of D-cells was 15.0 ± 11.8 in the antrum and 16.0 ± 11.9 in the gastric body. Similarly, for those with nausea and/or vomiting, the values were 16.0 ± 11.9 and 6.9 ± 5.9, and for those with diarrhea, the values were 16.4 ± 11.9 and 8.8 ± 7.7, respectively. The number of D-cells in the body was significantly lower than that in the antrum for all symptoms (*p* < 0.05). HPF, high-power field.

The mean number of D-cells per HPF in the gastric antrum and body was analyzed based on patient symptoms (Figure 2). Children with substernal pain had 15.8 ± 12.8 D-cells per HPF in the gastric antrum and 7.1 ± 6.7 D-cells in the gastric body. In those with abdominal pain, the number of D-cells was 15.0 ± 11.8 in the antrum and 16.0 ± 11.9 in the body. Similarly, for those with nausea and/or vomiting, the values were 16.0 ± 11.9 and 6.9 ± 5.9, and for those with diarrhea, the values were 16.4 ± 11.9 and 8.8 ± 7.7, respectively. The number of D-cells in the body was significantly lower than that in the antrum for all symptoms (*p* < 0.05).

### 3.3. Number of D-cells per HPF according to reflux status

**Figure 3:**
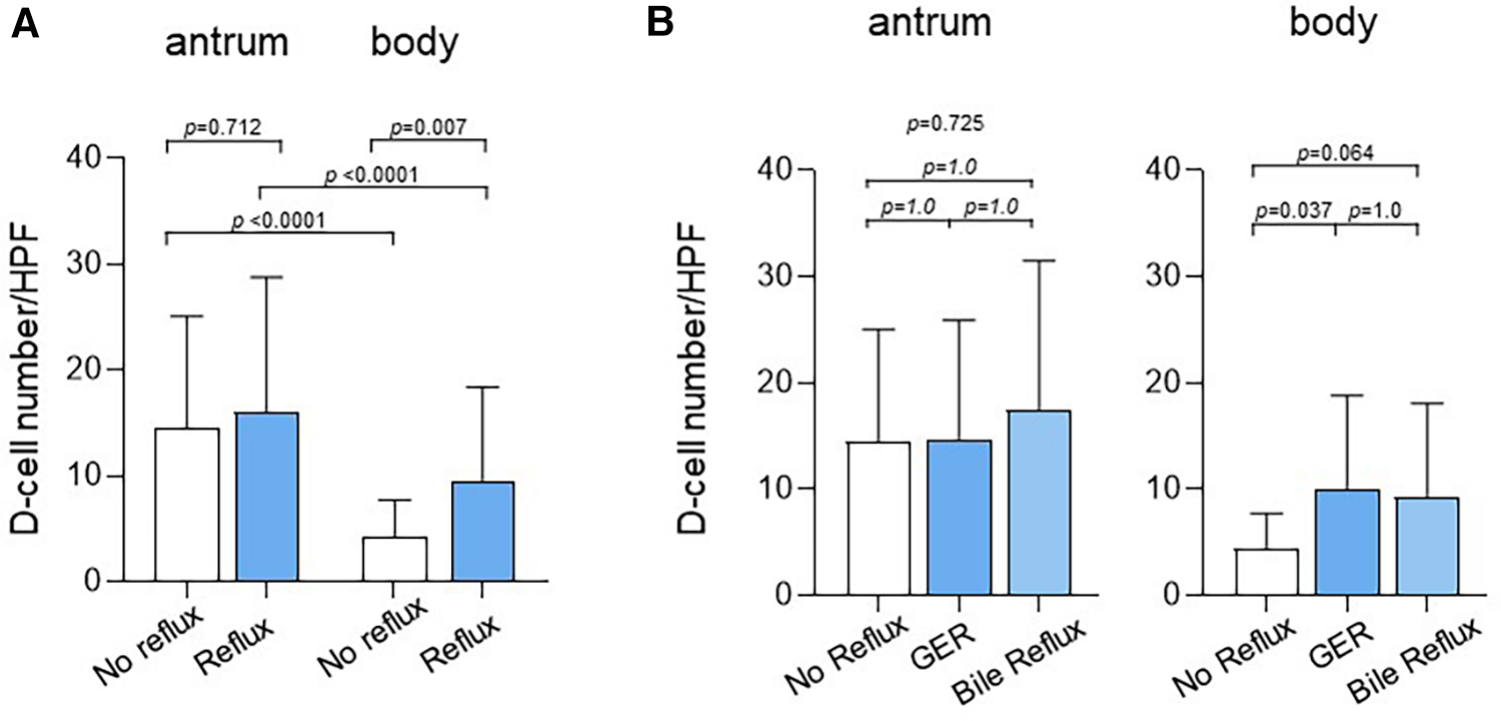
Number of D-cells in the antrum and body according to reflux status. (**A**) Children with reflux had a significantly higher mean number of D-cells (9.6 ± 8.8) in the gastric body than that in children without reflux (4.3 ± 3.4, *p* = 0.007). Children with reflux also had a higher number of D-cells in the gastric antrum (16.1 ± 12.7) than that in those without reflux (14.5 ± 10.6); however, the difference was statistically insignificant (*p* = 0.712). (**B**) The number of D-cells in the body was 4.3 ± 3.4 in children without reflux, 10.0 ± 8.8 in children with GER (*p* = 0.037), and 9.2 ± 8.9 in children with bile reflux (*p* = 0.064). GER, gastroesophageal reflux.

The number of D-cells per HPF was evaluated based on reflux status to investigate the association between reflux disease and somatostatin levels (Figure 3A). Children with reflux had a significantly higher mean number of D-cells (9.6 ± 8.8) in the gastric body than that of those without reflux (4.3 ± 3.4, *p* = 0.007). Children with reflux also had a higher number of D-cells (16.1 ± 12.7) in the gastric antrum than that of those without reflux (14.5 ± 10.6); however, this difference was statistically insignificant (*p* = 0.712).

The number of D-cells was also compared between children with GER and those with bile reflux (Figure 3B). The number of D-cells in the gastric body was 4.3 ± 3.4, 10.0 ± 8.8, and 9.2 ± 8.9 in children without reflux, those with GER, and those with bile reflux, respectively, showing a significant difference between those without reflux and those with GER.

### 3.4. Number of D-cells per HPF according to *H. pylori* infection

**Figure 4:**
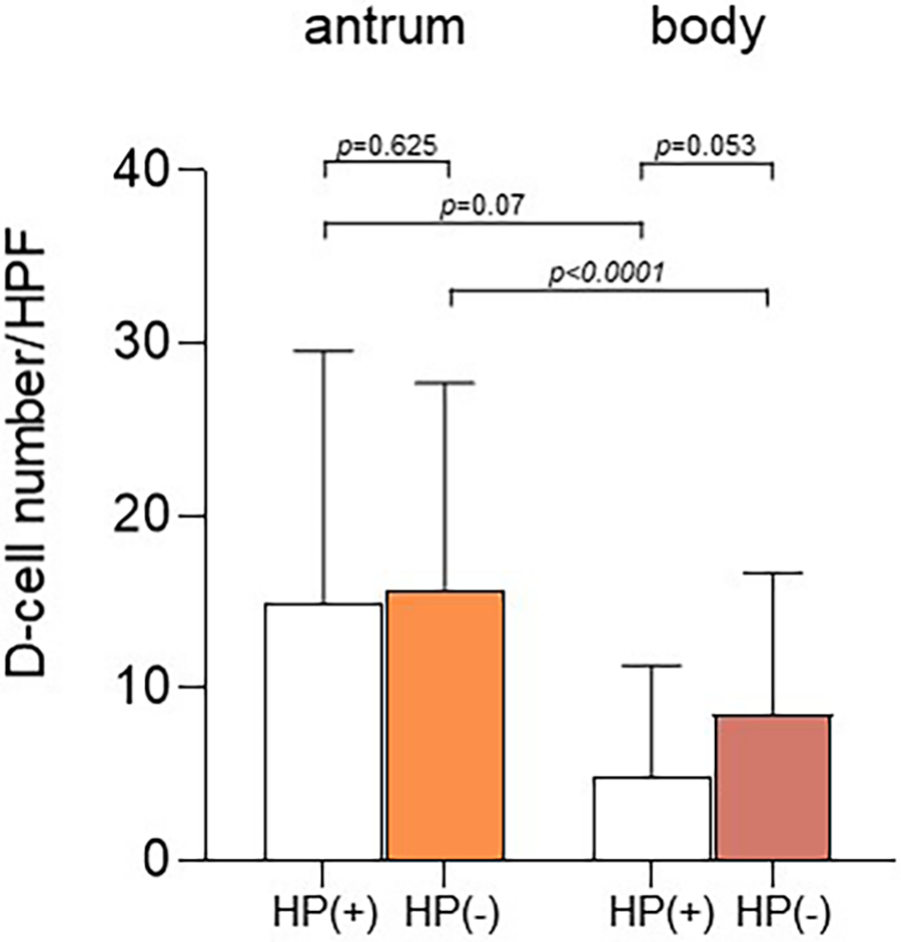
Number of D-cells according to *Helicobacter pylori* infection. The number of D-cells in the antrum was 14.9 ± 11.9 in *H. pylori*-positive children and 15.8 ± 14.7 in *H. pylori*-negative children. In the gastric body, the number of cells was 4.9 ± 6.5 in *H. pylori*-positive children and 8.5 ± 8.2 in *H. pylori*-negative children (*p* = 0.053). The number of D-cells in the antrum was significantly different from that in the body in both *H. pylori*-positive (*p* = 0.07) and *H. pylori*-negative children (*p* < 0.0001).

We compared the mean number ± SD of D-cells per HPF in the gastric antrum and body between *H. pylori*-positive and *H. pylori*-negative children (Figure 4). The number of D-cells in the antrum was 14.9 ± 11.9 in *H. pylori*-positive children and 15.8 ± 14.7 in *H. pylori*-negative children. In the gastric body, the number was 4.9 ± 6.5 in *H. pylori*-positive children and 8.5 ± 8.2 in *H. pylori*-negative children. The number of cells in the antrum was significantly different from that in the body in both *H. pylori*-positive and *H. pylori*-negative children.

## 4. Discussion

The basic pathophysiological stimuli in the development of UGI diseases include acid secretion, GI motility, circulation, and mucosal barrier dysfunction. Acid secretion by parietal cells promotes the digestion of proteins and the absorption of micronutrients and decreases the risk of GI infection (16). Inadequate gastric acid secretion can lead to the malabsorption of nutrients and increase susceptibility to GI infections. However, excessive acid exposure can cause severe mucosal damage, leading to mucosal bleeding or perforation. Therefore, the control of acid secretion is crucial in the treatment of UGI diseases. Somatostatin is the main inhibitor of acid secretion in the stomach. D-cells, as the major gastric endocrine cells, play a crucial role in regulating acid secretion in the stomach (17). Acid production in parietal cells is regulated through neural, hormonal, and paracrine mechanisms, as well as intracellular pathways that control proton pumps in parietal cells. Neuronal stimulation through acetylcholine, hormonal control via gastrin, and paracrine stimulation by histamine released from ECL cells induce acid production (18). Several studies have examined the relationship between somatostatin use and UGI diseases. Patients with gastric ulcers or gastritis exhibit significantly lower numbers of somatostatin-producing D-cells in the antrum (19). D-cells are located in proximity to target cells and strongly suppress gastric acid, gastrin, and histamine secretion (16).

*H. pylori* infection also influences gastric secretions. *H. pylori*-induced gastric inflammation inhibits gastric acid secretion via somatostatin-mediated pathways to favor the survival of *H. pylori* in the presence of strong acids in the gastric mucosa (16). Conversely, some studies have revealed that *H. pylori* infection reduces the D-cell count in the antral mucosa (20–24). In an animal model, acute *H. pylori* infection suppressed acid secretion by intramural sensory neural activation, somatostatin enhancement, and histamine inhibition (25). These results prompted us to investigate *H. pylori*-associated D-cell distribution in the stomach.

Another major physiological effect of somatostatin is the regulation of GI motility. As mentioned in the introduction section, somatostatin regulates gastric motility, delays gastric emptying, increases stomach volume, suppresses acid and pepsin secretion, inhibits gastrin release, and inhibits LES pressure. A study among healthy volunteers showed that intravenous somatostatin infusion increases the LES tone, contraction amplitude, and velocity of the esophageal body, which is mediated by a direct effect and central nervous system action (26). Another study in volunteers showed that intravenous somatostatin infusion prevents postprandial reduction in LES pressure and inhibits swallowing-induced LES relaxation but does not affect transient LES relaxation (27). The clinical manifestation of delayed gastric emptying in patients undergoing pancreaticoduodenectomy is significantly decreased by somatostatin prophylaxis (28). A similar immunohistochemical staining study of somatostatin D-cells in adult patients with DGR showed a decrease in the D-cell count in the gastric antrum and body (29). The authors of this study concluded that DGR inhibits somatostatin release. In an animal study, DGR suppressed serum somatostatin levels, but this suppression was not observed in cases of bile diversion (30). However, we believe that no conclusion can be drawn because the number of cells could not be analyzed before the disease developed. Few pediatric studies have investigated the effects of somatostatin on GI motility. A study among children with chronic GI disorders showed that parenteral somatostatin administration delays gastric emptying during fasting and intestinal phase II movements (31).

The current study aimed to investigate whether a change in GI peptide levels may be a fundamental trigger or an inducing factor of reflux disease. Most studies on GI peptides were performed 2–3 decades ago. However, very few studies have been conducted on pediatric GI diseases. We attempted to determine whether the amount of tissue somatostatin is related to the development of reflux disease. In a previous study among a few children, the number of D-cells did not significantly differ according to symptoms and endoscopic findings; however, it was fewer in the gastric body of children with a current *H. pylori* infection (32). In the present study, the D-cell count was higher in the gastric body of children with DGR and was significantly lower in *H. pylori*-infected children, which is similar to the findings in our previous study and other groups (33). This may explain why stomach motility is decreased in DGR and why patients with *H. pylori* infection occasionally have motility disorders.

This study has the following limitations: G-cell immunohistochemistry staining results were inconsistent in our pilot study; therefore, we could not compare G-cells and D-cells. Furthermore, the number of D-cells was only counted during active disease and not before the onset of the disease or symptom. Therefore, we cannot ascertain whether the differences in counts observed were the causes or results of the GI pathology. Based on our results, we assumed that somatostatin peptide secretion might be the major pathophysiological pathway of GI reflux diseases. It is difficult to obtain consent for follow-up gastrofibroscopy after symptomatic improvement in children with these diseases, and it is even more difficult to obtain GI biopsies. If these limitations are addressed, understanding the changes in D-cell counts before and after diagnosis of the diseases will be easier. Further research is needed to establish causality and elucidate the detailed mechanisms of the role of gastric D-cells in these diseases.

### 4.1. Conclusion

In summary, this study investigated the relationship between somatostatin-secreting gastric D-cells and GI diseases in pediatric patients. The number of D-cells in the gastric body decreased in *H. pylori*-positive children and significantly increased in children with reflux disease. This suggests that the number and distribution of gastric D-cells may vary according to GI diseases. Therefore, somatostatin peptide secretion may be a key target for the treatment of pediatric GI diseases, especially reflux disease.

## Data Availability

The original contributions presented in the study are included in the article/Supplementary Material, further inquiries can be directed to the corresponding authors.
